# New findings on clinical experience on surface‐guided radiotherapy for frameless non‐coplanar stereotactic radiosurgery treatments

**DOI:** 10.1002/acm2.14510

**Published:** 2024-09-17

**Authors:** Patricia Sánchez‐Rubio, Ruth Rodríguez‐Romero, María Pinto‐Monedero, Luis Alejo‐Luque, Jaime Martínez‐Ortega

**Affiliations:** ^1^ Medical Physics Department Hospital Universitario Puerta de Hierro Majadahonda Madrid Spain

**Keywords:** AlignRT, frameless non‐coplanar SRS, intrafraction control, open‐face mask, SGRT

## Abstract

**Purpose:**

The aim of this study was to assess the accuracy of a surface‐guided radiotherapy (SGRT) system for setup and intra‐fraction motion control in frameless non‐coplanar stereotactic radiosurgery (fSRS) using actual patient data immobilized with two different types of open‐faced masks and employing a novel SGRT systems settings.

**Methods and materials:**

Forty‐four SRS patients were immobilized with two types of open‐faced masks. Sixty lesions were treated, involving the analysis of 68 cone‐beam scans (CBCT), 157 megavoltage (MV) images, and 521 SGRT monitoring sessions. The average SGRT translations/rotations and 3D vectors (MAG‐Trasl and MAG‐Rot) were compared with CBCT or antero‐posterior MV images for 0° table or non‐coplanar beams, respectively. The intrafraction control was evaluated based on the average shifts obtained from each monitoring session. To assess the association between the SGRT system and the CBCT, the two types of masks and the 3D vectors, a generalized estimating equations (GEE) regression analysis was performed. The Wilcoxon singed‐rank test for paired samples was performed to detect differences in couch rotation with longitudinal (LNG) and lateral (LAT) translations and/or yaw.

**Results:**

The average SGRT corrections were smaller than those detected by CBCT (≤0.5 mm and 0.1°), with largest differences in LNG and yaw. The GEE analysis indicated that the average MAG‐Trasl, obtained by the SGRT system, was not statistically different (*p* = 0.09) for both mask types, while, the MAG‐Rot was different (*p* = 0.01). For non‐coplanar beams, the Wilcoxon singed‐rank test demonstrated no significantly differences for the corrections (LNG, LAT, and yaw) for any table rotation except for LNG corrections at 65° (*p* = 0.04) and 75° (*p* = 0.03) table angle position; LAT shifts at 65° (*p* = 0.03) and 270° (*p* < 0.001) table angle position, and yaw rotation at 30° (*p* = 0.02) table angle position. The average intrafraction motion was < 0.1 mm and 0.1° for any table angle.

**Conclusion:**

The SGRT system used, along with the novel workflow performed, can achieve the setup and intra‐fraction motion control accuracy required to perform non‐coplanar fSRS treatments. Both masks ensure the accuracy required for fSRS while providing a suitable surface for monitoring.

## 1 INTRODUCTION

The application of surface‐guided radiotherapy (SGRT) in clinical practice is increasing^1^, ^2^, ^3^ and includes patient positioning^4^, ^5^, ^6^, ^7^ respiratory control,^8^, ^9^, ^10^, ^11^ improved patient safety,^12^, ^13^, ^14^, ^15^ and even radiation dose estimation using Cherenkov light emission.^16^, ^17^, ^18^, ^19^, ^20^ In intracranial locations,^21^, ^22^ SGRT provides additional patient comfort, in conjunction with frameless systems, allowing the use of open masks during treatment. Furthermore, this enables positioning verification and intrafraction control for non‐coplanar beams, which are common in frameless stereotactic radiosurgery (fSRS) to maximize organ‐at‐risk (OAR) protection and diversify radiation beam incidences.

The fSRS technique requires precise localization of the treatment volume because of the high doses involved in a single fraction delivery. The SGRT systems employed for fSRS are recommended to be specifically calibrated to achieve a global accuracy of < 1 mm.^23^ Additionally, for effective real‐time monitoring, it is recommended that the instantaneous fluctuations inherent to SGRT equipment should not exceed 0.5 mm.^24^

SGRT for fSRS has seen an increasing interest. While the accuracy achieved by the SGRT systems has mainly been documented in phantoms,^25^, ^26^, ^27^, ^28^, ^29^, ^30^ the evaluation of these systems in conjunction with the use of non‐coplanar couch angles has not been sufficiently evaluated for clinical efficacy in terms of positioning and intrafraction monitoring accuracy based on real patient data.

Covington et al.^31^ reported the use of a SGRT system for intrafraction control at non‐zero couch angle based on a large cohort of patient data, using 1 mm threshold and manual control of the beam delivery, but only magnitude of the translational offset was evaluated. On the other hand, Lai et al.^32^ exclusively evaluated the accuracy of a SGRT system for patient set‐up (translations and rotations) for a limited range of couch rotation, but no evaluation was made in relation to the intrafraction control. Moreover, the recent work of Covington et al.,^33^ who evaluated the performance of a new commercial surface imaging, the couch angles analyzed were limited to 0°, 45°, 90°, 315°, and 270° since patients were treated with HyperArc.

The aim of this study is to evaluate the accuracy of a SGRT system in fSRS for initial positioning and intrafraction control, using data from real patients treated by any non‐coplanar beams, with a new SGRT sytems settings. Additionally, not only the translational offsets at non‐zero couch are evaluated, but also the rotational offsets. The feasibility of two types of open masks is also assessed.

## 2 METHOD AND MATERIALS

This study analyzed our clinical experience with the SGRT system AlignRT (VisionRT Ltd, London, UK). The system, equipped with high‐definition cameras, was installed in early 2020 on a TrueBeam STx linear accelerator manufactured by Varian Medical Systems, Inc. This linear accelerator is further equipped with a high‐definition multileaf collimator, a *Perfect Pitch* robotic couch with six degrees of freedom (DoF), and a cone‐beam computed tomography (CBCT) image guidance system.

### 2.1 Patient samples

**FIGURE 1:**
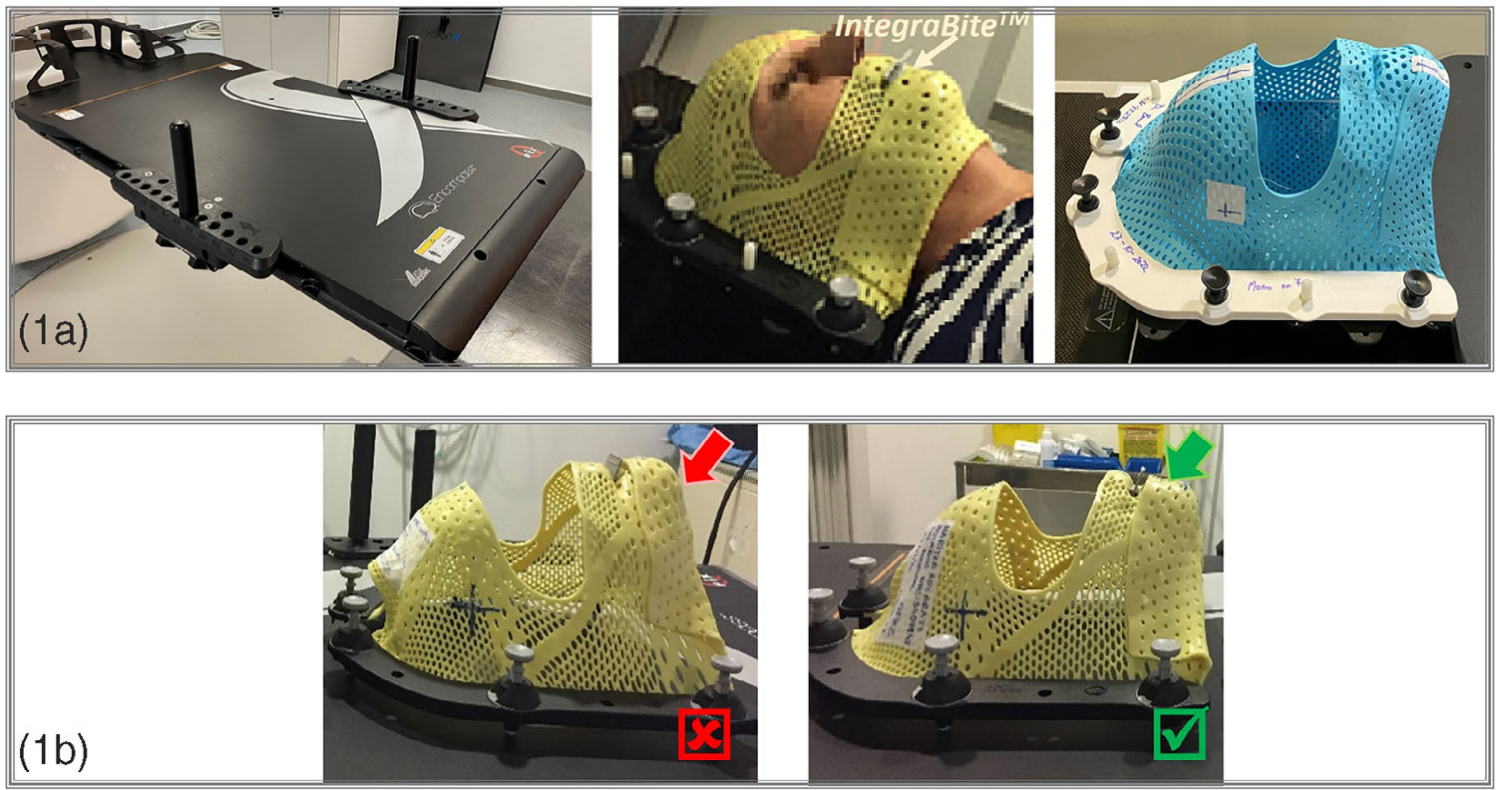
Immobilization system and open face masks. (a) The image on the left shows the Encompass SRS standalone device with the hand grip array. The yellow mask is the Encompass SRS Fiberplast® mask, and the blue one corresponds to the thermoplastic mask Klarity Blue. (b) The panels show how the chin does not occlude the open area of the mask when the neck of the patient is not placed in hyperextension.

A retrospective analysis between June 2020 and August 2022 was conducted on 44 patients treated with fSRS, corresponding to a total of 60 cranial locations (0.05–5 cm^3^, 12−18 Gy) (Table 1). Of these, 18 cases were immobilized using the Qfix Encompass System along with the Encompass SRS Fibreplast mask (Avondale, Pennsylvania, USA), and 42 cases were immobilized using the Klarity Blue thermoplastic mask (Klarity Medical Products USA, Heath, Ohio, USA), compatible with the Encompass immobilization system (Figure 1a). Both masks were created using the IntegraBite bite block (Avondale, Pennsylvania, USA) to ensure upper jaw positioning reproducibility. To maximize the monitoring area and prevent loss of precision by the SGRT system,^27^ patients were simulated with their chins slightly flexed toward the chest (Figure 1b) as opposed to the typical hyperextension position, which reduces the available surface for monitoring when the craniocaudal axis of the patient was parallel to the camera line of sight. The Klarity Blue mask allowed for a slightly larger open region compared with that of the Encompass SRS Fibreplast mask. The opening of the mask extended longitudinally from the hairline of the patient to the middle of the philtrum and laterally to the level of the anterior auricular muscles. Additionally, three radio‐opaque markers were placed on the mask at the nasion level.

**TABLE 1:** Main characteristics of the 60 locations treated in single session.

Pathology	Number of cases	Mean volume (cm^3^)	Volume range (cm^3^)	Prescription dose (Gy)
AVM	1	4.84	–	17
Meningioma	1	0.6	–	13
Acoustic Neurinoma	10	0.62	(0.05–2.73)	12/18
Brain metastases	48	0.67	(0.05–4.84)	12/13/18

Volumetric‐modulated arc therapy was employed in 55 locations, conformal arc therapy in three other locations because of the small volume of the CTVs (0.21, 0.08, and 0.05 cm^3^), and dynamic intensity‐modulated radiation therapy for two locations where patients had previous irradiations. An average of four arcs were used per target, where a patient customized couch angle was selected for each arc. For patients with multiple metastases, a separate isocenter was used for each lesion.

### 2.2 The SGRT system

The AlignRT system uses three positioning optical devices (PODs) located on the ceiling. Each POD contains two stereoscopic vision cameras and a projector that emits a pattern of structured light onto the patient's skin. The reflected pattern is captured by the cameras, and the software reconstructs a real‐time image of the skin surface. AlignRT performs rigid registration^34^, ^35^ for a region of interest (ROI) between the reference surface and the acquired surface, enabling the determination of relative displacements between the two surfaces in six DoF, known as real‐time delta (RTD).

The manufacturer recommends daily verification of the integrity of the three PODs (all pointing to the radiation isocenter of the accelerator), monthly calibration and alignment check between the optical isocenter of AlignRT, and the gantry radiation isocenter. Paxton et al.,^36^ Wiant et al.,^27^ Covington et al.,^37^ and Zhang et al.^38^ have shown that improvements in software and calibration procedures can enhance the tracking accuracy for non‐zero table angles, reducing false positives for head motion at these angles. Our center implemented a quality assurance (QA) program to ensure sufficient accuracy for SRS. The QA program consisted of daily checks to verify that the consistency between the PODs was root mean square (RMS) < 0.5 mm; weekly checks using the Varian IsoCal tool^39^ were also conducted to ensure the alignment of the radiation isocenter with the MV and kVCBCT imaging systems, with a tolerance of < 0.5 mm per projection axes (X, Y). A cubic phantom containing five radio‐opaque spheres, provided by VisionRT, was used to verify deviation between the optical isocenter of SGRT and the MV isocenter, aiming for a difference per axes < 0.1 mm and 0.1°. Additionally, the accuracy of AlignRT with table isocentric rotation was verified by performing the Hancock‐Wiston‐Lutz test with the sphere located in the center of the cube, with a tolerance of ≤0.5 mm and ≤0.5°. If any of these checks exceeded the specified tolerances, the imaging system isocenter was recalibrated based on the IsoCal results using the offsets between the position of the imager panels and the treatment isocenter to generate corrections consisting of physical panel shifts in the lateral and longitudinal direction, and/or the AlignRT isocenter was realigned with the MV isocenter using a Winston‐Lutz type process with the cubic phantom.

**FIGURE 2:**
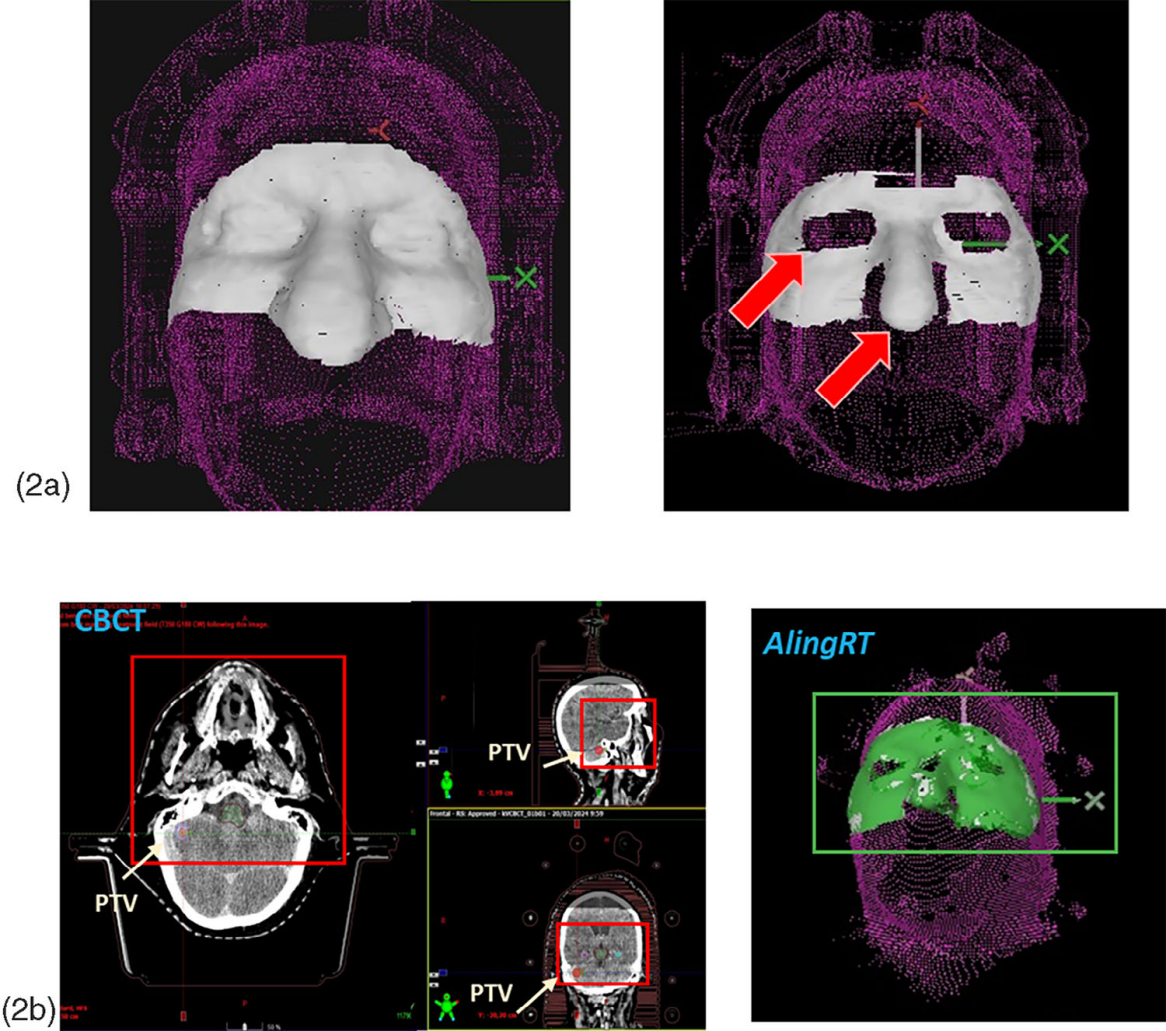
ROI used for SGRT and CBCT. (a) The contoured ROI for surface monitoring included the forehead, eyes, nose, and temporal bones. If the patient blinks too much or has very deep breathing, the eyelids and nose flaps are excluded. (b) A ROI centered on the target site and with similar size to the registration area used in AlignRT is employed to register CBCT with the planning CT images.

The “Intracranial SRS*”* protocol was selected to maximize the resolution of the SGRT point mesh (minimum 3D resolution of 10 000 points, with a surface monitoring frame rate of 7−10 frames per second). The tolerance thresholds for beam interruption during treatment were set at 0.3 mm for translations and 0.3° for rotations. Furthermore, to minimize interruptions arising from surface detection fluctuations caused by camera occlusions, we opted for a 1‐s interval for the “Maximum Misalignment Time” parameter. Within this duration, displacements and rotations beyond the previously mentioned tolerances were permissible. The contoured ROI is shown in Figure 2a.

### 2.3 Clinical workflow for SRS

**FIGURE 3:**
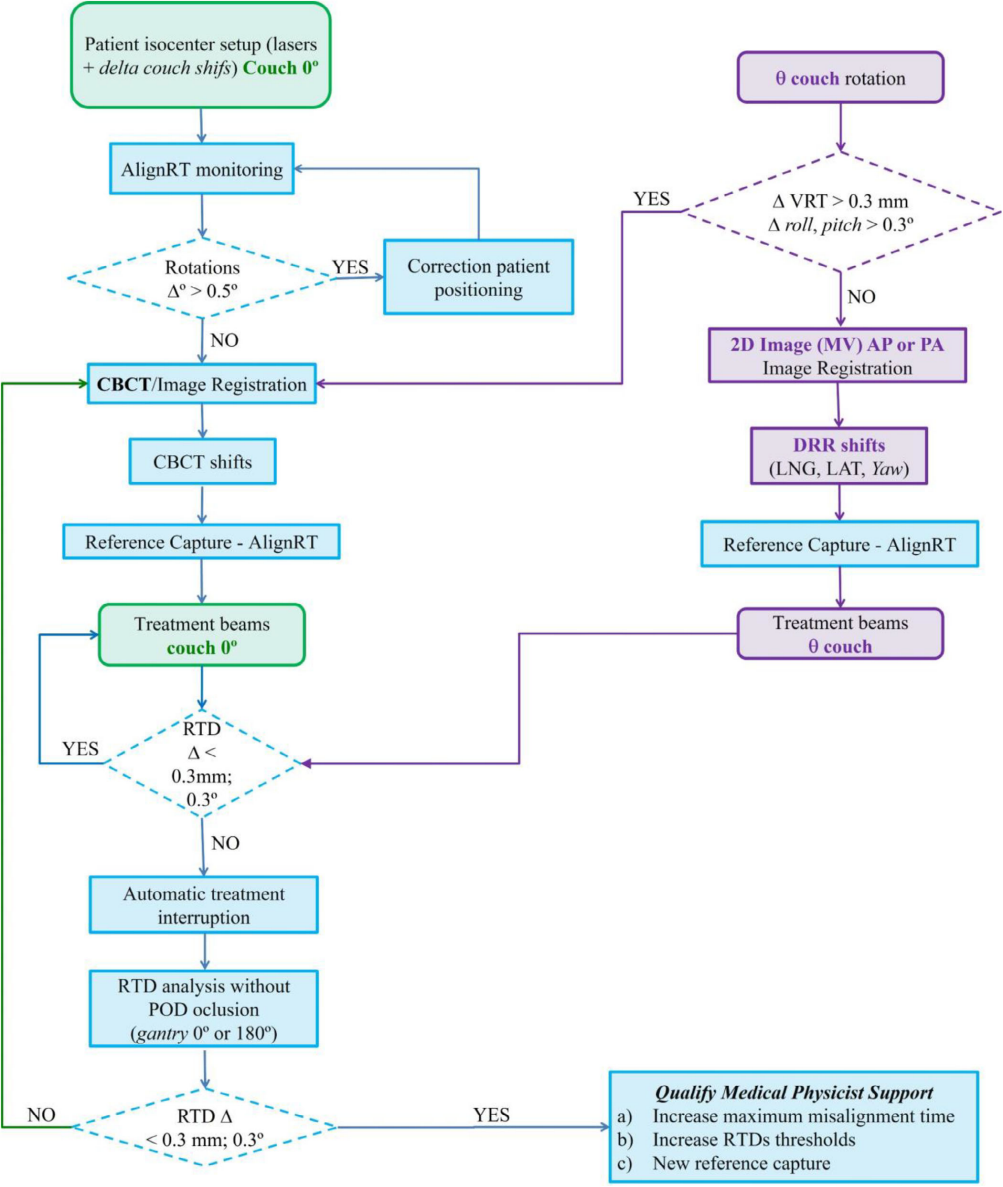
Treatment workflow. SGRT/IGRT workflow for frameless SRS treatment. θ, couch rotation angle; RTD, real‐time deltas.

The clinical workflow for fSRS in our facility is shown in Figure 3. First, the patient was positioned at the isocenter after aligning the radio‐opaque markers of the mask with the room lasers and automatically executing the Delta Couch shifts recorded in ARIA. Subsequently, monitoring with AlignRT was performed during treatment set‐up, and patients’ heads were adjusted to correct rotations > 0.5° obtained by comparing the real‐time surface with the DICOM surface of the patient exported from the treatment planning system to AlignRT.

Thereafter, a CBCT scan was acquired and registered with the planning CT, using a ROI centered on the target site (Figure 2b). After applying corrections in all six DoF, a new reference surface was acquired and the arcs/fields with 0° couch angle or close to 0° (± 10°–15°) was treated afterwards. Before each non‐coplanar arc, antero‐posterior (AP) MV images were acquired, and after registration, longitudinal (LNG), lateral (LAT), and yaw corrections were applied. Finally, a new reference surface was captured.

Treatments were delivered by gating the beams via AlignRT and were only interrupted when RTDs exceeded thresholds > 0.3 mm for translations and/or 0.3° for rotations for > 1 s (*maximum misalignment time)*. The choice of 1 s for the *maximum misalignment time* is not only the manufacturer's recommendation to avoid beam interruption due to false positive caused by inherent temporal fluctuations of the SGRT systems, but also the shortest delay time available in the application. To determine whether the interruption was due to actual patient movement, gantry occlusion, or improper propagation of the reference ROI for a specific table position, the gantry was set to 0°/180° and/or the table to 0°. Genuine patient movement was confirmed if the RTDs continued to exceed thresholds without gantry occlusion and/or couch rotation. In such a case, a new CBCT scan was performed, followed by the appropriate corrections, a new reference surface was acquired, and the treatment was resumed.

The system remained powered to prevent drift errors of approximately 0.3 mm, attributed to the time needed for the cameras in the SGRT system to attain optimal temperature.^40^, ^41^ Also, AlignRT monitoring was activated from the moment the patient was positioned on the table to warm‐up the AlignRT system in the RTD mode to eliminate the baseline‐drift error, which produces an error of ∼0.3 mm^40^ due to the thermal heat in the camera system, but stabilizes after 5 to 10‐min RTD mode.

### 2.4 Data analysis

We evaluated 60 locations, which corresponded to 44 patients, and involved the analysis of 68 CBCT scans, 157 MV images, and 521 SGRT monitoring sessions.

The accuracy of AlignRT for initial positioning at 0° table angle was evaluated by comparing the RTD with the corrections obtained after performing CBCT scans for LNG, LAT, and vertical (VRT) translations, and the 3D displacement vector (MAG Trasl), as well as roll, pitch, yaw rotations, and 3D rotation vector (MAG Rot = √ Pitch^2^ + Roll^2^ + Yaw^2^). This magnitude (MAG Rot), which lacks physical meaning, was calculated to allow for comparison with other studies.^40^ For beams with table rotation, the LNG and LAT translations, and yaw rotation obtained from AP MV image were compared with those obtained from AlignRT.

The intrafraction control variability achieved by AlignRT was evaluated for translations and rotations based on the average RTDs obtained from each monitoring session.

Collected data were considered to be quantitative continuous variables, and their distributions were evaluated using mean, standard deviation (SD), median, interquartile range (IQR), and 95% confidence interval (CI). The 95% CI (alpha = 0.05) was estimated as ± 1.96 * SD/√n (n: sample size) when *n* > 30, or as ± Student's *t*‐test 0.05/2*SD/√n if *n* < 30. Before selecting the appropriate statistical test for hypothesis testing, the normality of the distribution across MAG Trasl and MAG Rot, obtained from AlignRT and CBCT for both types of masks, was checked by the Shapiro‐Wilk test. In cases where *p* < 0.05, the measurements were non‐normally distributed. In addition, Levene's test was performed to statistically evaluate the equality of variance between the 3D vectors for each mask type, obtained through CBCT or AlignRT. In cases where *p* < 0.05, the variance was regarded as unequal. Although the 3D vectors met the homoscedasticity condition, they were not normal, so a generalized estimating equations (GEEs) regression^42^ analysis was performed to assess the association between the positioning systems, the two types of masks, and the 3D vectors. This analysis takes into account that for each patient, the 3D vectors are obtained twice, one by CBCT and another one by AlignRT. Dependent variable was the 3D vectors. The link function was identity and the covariance structure was exchangeable. The effect size in the 3D vectors depending on the type of mask or the positioning systems were estimated along with their corresponding 95% CI. Differences were considered significant if *p* < 0.05.

Additionally, the Wilcoxon signed‐rank test for paired samples was conducted to detect any table angle where LNG and LAT translations and/or yaw rotation significantly differed, regardless of the type of mask used. Again, differences were considered significant if *p* < 0.05. In order to determine whether two techniques (AlignRT vs. MV) agreed sufficiently well, Bland and Altman (B&A) plots was done for those couch angle where the Wilcoxon singed‐rank test for paired samples was statistically significant (*p* < 0.05). In the B&A plot, the difference of the two paired measurements was plotted against the average of the two measurements. Also, 95% limits of agreement, mean difference ± 1.96 its SD, was represented, which would tell us how far apart measurement by the two techniques were likely to be for most locations. The B&A plot does not say whether the agreement is sufficient or suitable to use AlignRT or the MV image. It simply quantifies the bias and a range of agreement 95% the differences between one measurement and the other are included.

Statistical analysis was performed using data analysis software (Excel version 2021, Microsoft Corp, Redmond, Washington, USA). STATA (StataCorp.2023.Stata Statistical Software: Release 18. College Station, Texas, USA: StataCorp LLC) was used for the GEE approach.

## 3 RESULTS

Table 2 displays the mean difference (Δ_SGRT—CBCT initial_) for the 60 locations, showing the largest mean differences in the LNG direction (0.9 ± 1.5 mm) (mean ± SD), regardless of the mask used. However, depending on the mask type, the largest Δ_SGRT—CBCT initial_ was found for pitch rotation. The GEE approach showed (Table 3) that the MAG Trasl was not statistically different for both mask types (*p* = 0.09), whereas the average MAG Trasl depended on the positioning system used to obtain it (*p* < 0.001), so that the MAG Trasl estimate was 0.61 mm larger when determined by AlignRT than by CBCT, with 95% CI (0.27–0.94 mm). However, for MAG Rot, the angular corrections were significantly different for each mask (*p* = 0.01), so that the MAG Rot estimate for the Qfix mask was −0.62° lower than for the Klarity mask, with a 95% CI (−1.10° to 0.14°), whereas it could not be concluded that there were different when determined using AlignRT or CBCT (*p* = 0.452).

**TABLE 2:** Translational and rotational differences between AlignRT and CBCT for 0° couch position for initial setup.

	VRT (mm)			LNG (mm)			LAT (mm)			MAG Trasl (mm)^a^		
∆_SGRT—CBCT intial_	Mean	SD	95% CI	Mean	SD	95% CI	Mean	SD	95% CI	Mean	SD	95% CI
Total (*n* = 60)	−0.2	1.1	(−0.6; 0.2)	0.9	1.5	(0.6; 1.1)	−0.2	0.9	(−0.5; 0.2)	0.6	1.3	(0.4; 0.9)
Qfix (*n* = 18)	0.4	0.9	(−0.3; 1.1)	1.4	1.4	(0.9; 1.9)	−0.1	1.0	(−0.5; 0.3)	0.9	1.5	(0.5; 1.4)
Klarity (*n* = 42)	−0.4	1.0	(−0.9; 0.1)	0.6	1.5	(0.4; 0.9)	−0.2	0.9	(−0.5; 0.1)	0.5	1.3	(0.2; 0.8)

	Pitch (°)			Roll (°)			Yaw (°)			MAG Rot (°)^a^		
∆_SGRT—CBCT intial_	Mean	SD	95% CI	Mean	SD	95% CI	Mean	SD	95% CI	Mean	SD	95% CI
Total (*n* = 60)	−0.1	1.3	(−0.4; 0.3)	−0.2	0.7	(−0.3; 0.0)	0.2	1.3	(−0.1; 0.6)	−0.2	1.5	(−0.5; 0.2)
Qfix (*n* = 18)	0.3	0.6	(0.0; 0.6)	0.0	0.7	(−0.4; 0.3)	0.2	0.7	(−0.2; 0.5)	0.1	0.7	(1.0; 0.3)
Klarity (*n* = 42)	−0.3	1.5	(−0.7; 0.2)	−0.2	0.7	(−0.4; 0.0)	0.2	1.5	(−0.2; 0.7)	−0.2	1.7	(−0.7; 0.3)

*n*: sample size (number of locations).

Abbreviation: SD, standard deviation.

^a^ Rotational 3D vector. Mag Trasl = √Vert^2^ + Long^2^ + Lat^2;^ Mag Rot = √Pitch^2^ + Roll^2^+ Yaw^2.^

**TABLE 3:** Relationships between the mean translational/rotational 3D vectors obtained by the SGRT/CBCT and type of mask.

		Qfix mask		Klarity mask			
		Median (mm)	IQR (mm)	Median (mm)	IQR (mm)	*p*‐value (Qfix vs. Klarity)	*p*‐value (SGRT vs. CBCT)
MAG Trasl	SGRT	3.04	1.86	3.14	1.45	0.09	< 0.001
	CBCT	2.08	1.13	2.42	1.37		
MAG Rot	SGRT	0.81	0.46	1.62	0.96	0.01	0.452
	CBCT	0.93	0.73	1.44	0.84		

Abbreviation: IQR, interquartile range.

**FIGURE 4:**
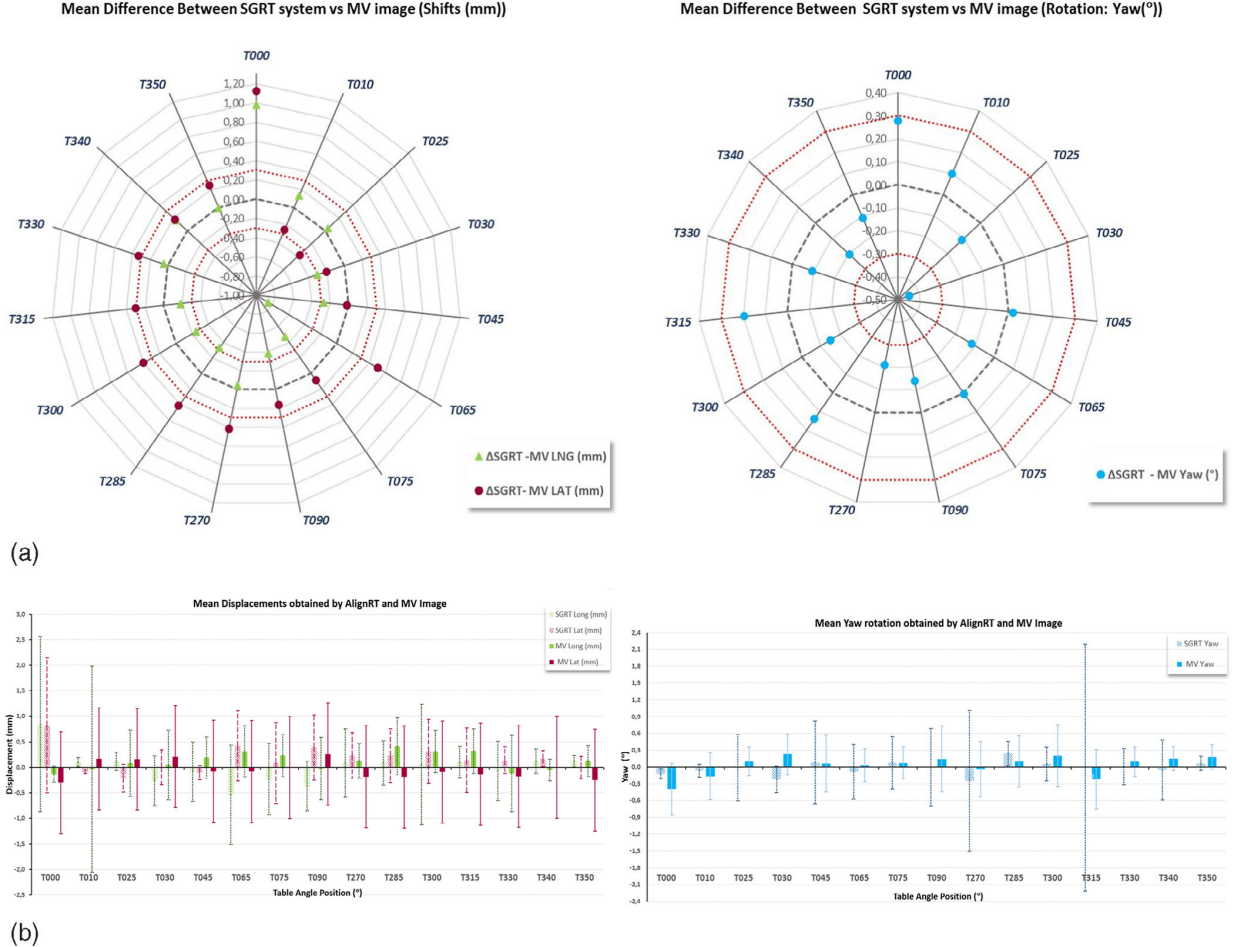
(a) Mean difference (SGRT—MV image) and (b) mean and standard deviation of longitudinal and lateral displacements (left) and rotation (right) detected by the SGRT system and the MV image for table angles used in the SRS treatments of the 60 locations analyzed.

The mean and the average differences between LNG and LAT translations and yaw angular correction detected by AlignRT and the AP MV image acquired before the irradiation of non‐coplanar beams are shown in Figure 4 [Statistical description of the differences (AlignRT—MV) is shown in Table S1]. For almost all table positions, the LNG, LAT, and yaw corrections detected by both systems differed less than ± 0.3 mm and ± 0.3°. The largest differences between both systems were found for the 0° table position, although the data only corresponded to an average of three patients: two for whom paired kV‐MV images were acquired, and one for whom intrafraction monitoring was performed using an MV image before irradiation of an arc at 0° table angle. For yaw correction, the greatest discrepancy between both systems was observed for table angles of 30°, with a maximum mean difference (± % 95 CI) −0.4° (−1.3° ‐ 0.4°) that was statistically significant (*p* = 0.02).

**FIGURE 5:**
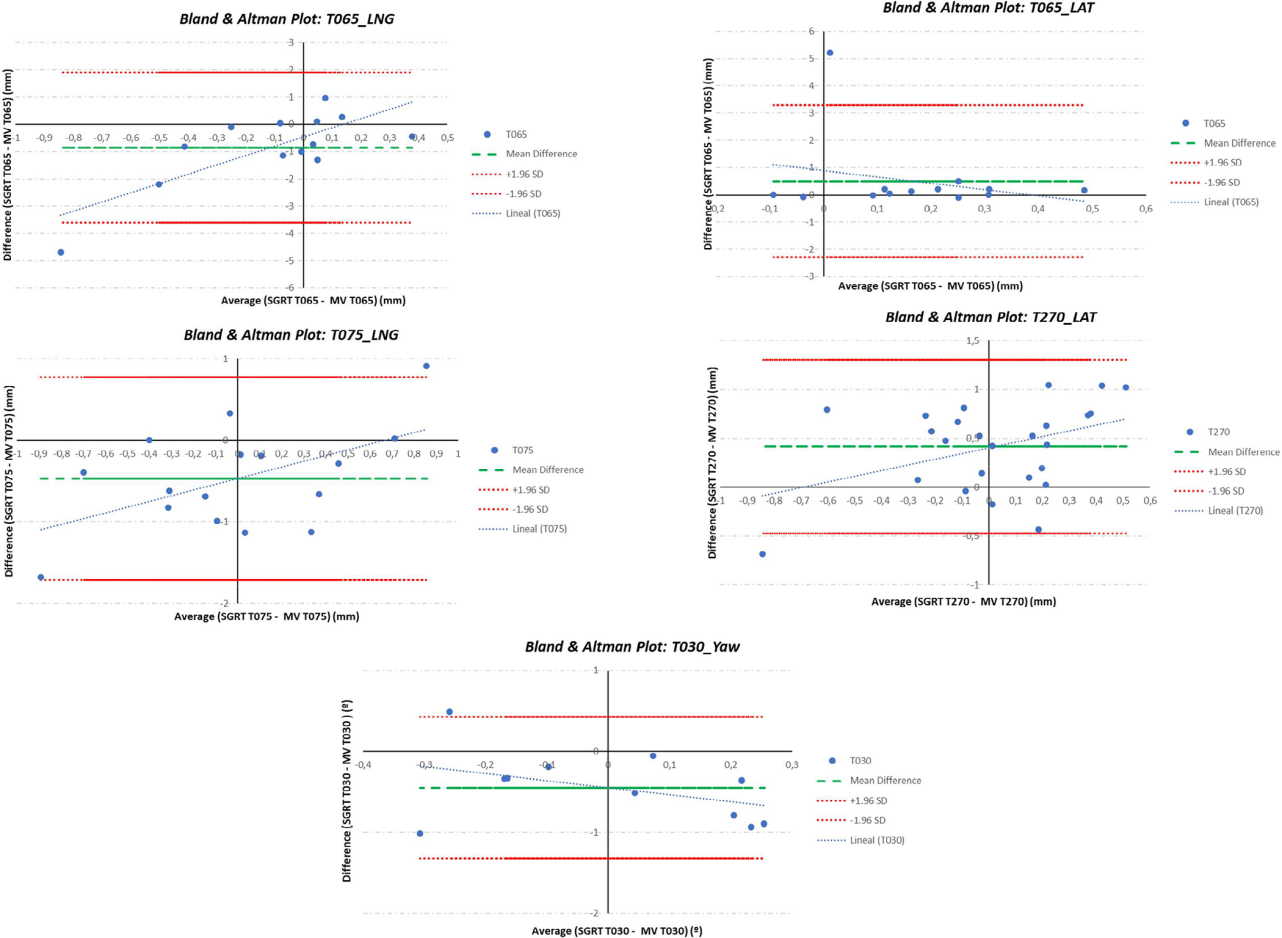
Bland and Atlman plots. The difference between AlignRT and the MV image is plotted against the average of the two measurements for those table angle position where the Wilcoxon singed‐rank test was statistically significant.

**FIGURE 6:**
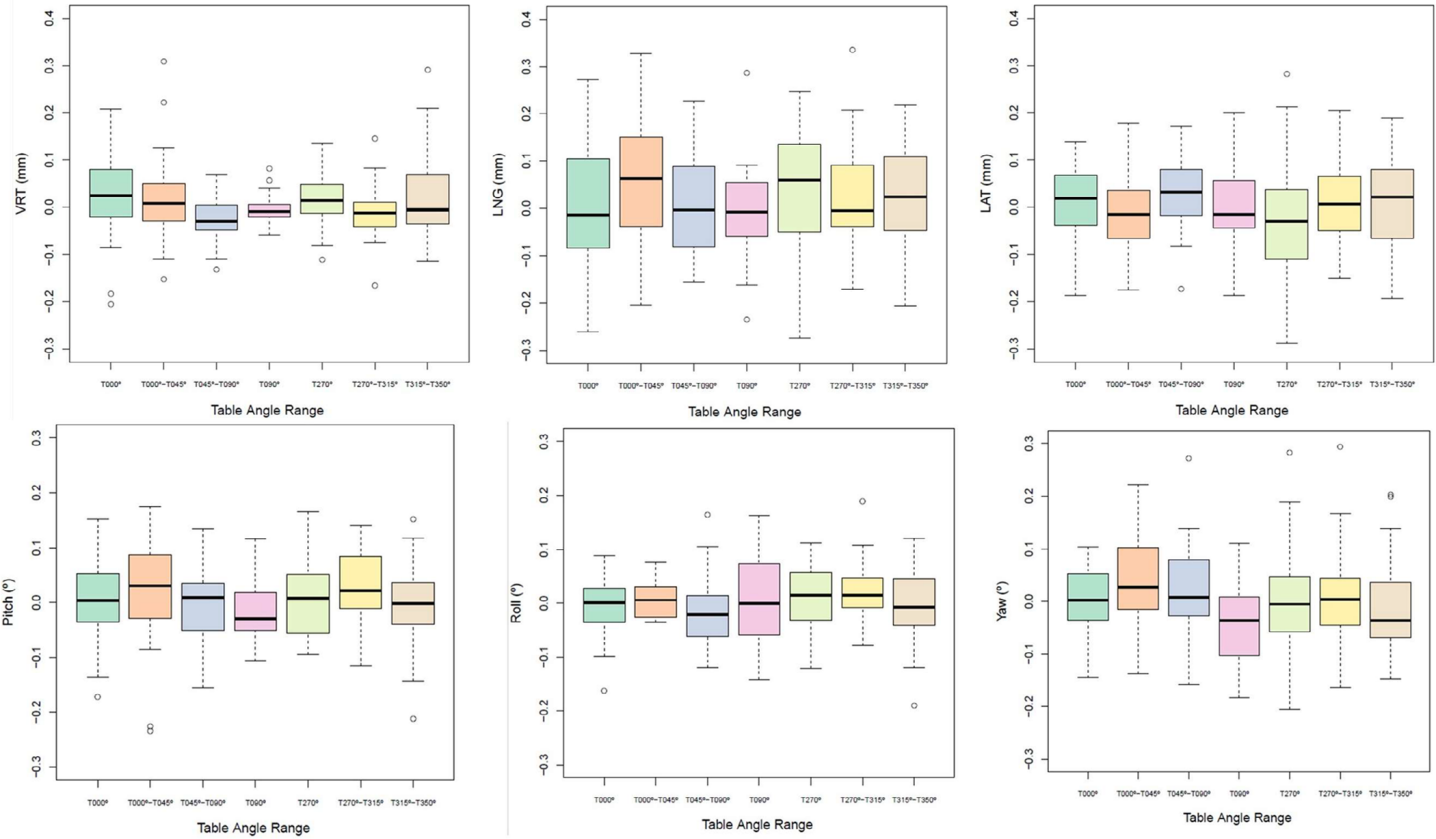
Intrafraction control for any couch rotation. Ranges of translations and rotations obtained with AlignRT during intrafraction control for different table positions.

The Wilcoxon singed‐rank test for paired samples showed that there were four table angle positions where the difference between both positioning systems was statistically significant: 65° for LNG (*p* = 0.04) and LAT (*p* = 0.03) corrections, 75° for LNG (*p* = 0.02), and 270° for LAT (*p* < 0.001) corrections. The B&A plots showed (Figure 5) that the mean differences with a 95% CI were −0.9 mm (−3.6 mm to 1.9 mm) and 0.5 mm (−2.3 mm to 3.3 mm) for LNG and LAT correction, respectively, for 65° table position; −0.5 mm (−1.7 mm to 0.8 mm) for LNG correction at 75° table angle; 0.4 mm (−0.5 mm to 1.3 mm) for LAT correction at 270° table angle. According to the B&A plots, AlignRT underestimates the LNG shift by −0.9, −0.5 mm at 65° and 75° table angle position, respectively; and the yaw rotation by −0.4° at 30° table angle; while it overestimates the MV image the LAT shift by 0.5  and 0.4 mm at 65°, 270° table angles, respectively. Since the difference between the corrections determined by AlignRT and MV image for LNG and LAT shifts at 65° table angle is not systematically positive or negative, so there is no systematic bias in either patient positioning system. However, AlignRT– MV difference for LNG at 75° table angle and for LAT at 270° table angle seems to show a trend in the bias, that is, a tendency for the mean difference to rise with increasing magnitude of the measurement (shown by the positive slope of the regression line), whereas the trend for the difference AlignRT—MV for yaw at 30° table angle seems to decrease when increasing the magnitude (shown by the negative slope of the regression line). The results of intrafraction control performed by AlignRT are shown in Figure 6. The average translations and rotations for any table angle were < 0.1 mm and 0.1°, respectively, with maximum average values of 0.06 mm and 0.04° for LNG axis and yaw rotation, respectively, for table positions within the T000° to T045° range. Furthermore, as depicted in Figure 6, it is precisely the LNG axis and yaw rotation where the highest variability was observed for any table position/angular range, compared with the rest of the translations and rotations. Outliers with values > 0.3 mm and 0.3° corresponded to cases where thresholds of 0.5 mm and 0.5° were mistakenly selected or where the established thresholds were repeatedly surpassed for 1 s without beam interruption. In addition, the intrafraction control was also analyzed considering the absolute value of the displacements and rotations. Although the average and median values were higher, the same conclusions could be drawn.

## 4 DISCUSSION

The MAG Trasl and MAG Rot vectors for the Klarity mask are larger than those for the Qfix mask (Table 3), possibly because of the slightly larger open area of the Klarity mask compared with that of the Qfix (3.14 mm and – 1.62° vs. 3.04 mm and – 0.81°). However, significant differences between the two masks were only found for the MAG Rot vector (*p* = 0.01). The main objective of the immobilization devices used in SGRT for SRS is not to completely immobilize the patient but to allow real‐time monitoring to detect when patient positioning needs to be readjusted, and thus, a larger open area provides a larger topographic area of the face, which is less susceptible to camera obstruction by the gantry and/or table rotation, and therefore achieves greater registration accuracy, as shown by Al‐Hallaq et al.^23^ Thus, although the Klarity mask allows for greater movement within it, it is equally suitable for use in frameless SRS with SGRT compared with the Qfix mask.

The accuracy of the AlignRT system for initial positioning at 0° table, evaluated as difference between the RTD and the corrections obtained after CBCT setup, showed the largest difference in the LNG direction (0.9 ± 1.5 mm) and the yaw angle (0.22° ± 1.34°). For lung and abdominal SBRT treatments, Heinzerling et al.^5^ also reported the LNG direction as the only significant difference between setups performed with SGRT or paired kV‐kV images However, Zhou et al.,^43^ who also used AlignRT for stereotactic radiotherapy (SRT) in 48 patients immobilized with a similar open‐face mask, reported the largest differences in the VRT and pitch directions, with a median difference of −0.5 mm and −0.2°, respectively. Lee et al.^40^ evaluated the setup differences between AlignRT and CBCT for 415 fractions corresponding to SRS with 269 patients and found that the largest dispersion of the difference distribution between both systems was in the LNG direction (1.0 ± 2.0 mm) and pitch rotation (0.0° ± 1.1°), with a minor correlation between pitch and LNG differences and roll and LAT differences of *r* = −0.44 and *r* = −0.29, respectively. They attributed this lack of correlation to the ambiguity of the SGRT system in estimating these corrections. Lee et al.^40^ also concluded that the main source of discrepancy between both systems was caused by the difference in the location of the ROIs used for registration in each system. In our center, despite trying to select a restricted or comparable ROI for CBCT registration (Figure 2b), the CBCT‐planning CT registration is based on a ROI that includes not only the anterior part of the head, but also the treatment volume, whereas the surface guidance registration was restricted to the ROI on the anterior surface. Therefore, greater setup differences will be detected by each system in treatments where the isocenter was further away from the ROI selected in AlignRT.^37^, ^40^ Tonkin et al.^44^ reported an error within 1 mm for isocenter depths ranging from 3 to 15 cm from a phantom surface in the SGRT system Catalyst^+^ HD (C‐Rad, Uppsala, Sweden).

Couch walkout will always contribute to displacements recorded in both CBCT/kV and SGRT systems; however, for TrueBeam accelerators, this drift is small and limited to between 0.1 and 0.7 mm.^38^, ^45^, ^46^, ^47^, ^48^, ^49^ These values are within the specifications outlined by AAPM's TG‐198 report^50^ for SRS. Nonetheless, any drift introduced by table movement was corrected when imaging was performed and a new reference surface was acquired. The mean displacements and rotations recorded in patients for different table rotations with the SGRT system were consequently < 0.5 mm and 0.3° (Figure 4b). Bry et al.^29^ reported the accuracy of Catalyst HD for non‐coplanar couch angles in comparison to the stereoscopic x‐ray system ExacTrac (BrainLab, Feldkirchen, Germany), using 3D anthropomorphic gel phantoms. They found that the discrepancies between the SGRT system and the x‐ray imaging were < 1 mm and < 0.5° in translational and rotational directions, respectively, concluding that Catalyst HD can be used for accurate positioning verification at non‐coplanar SRS treatment of multiple metastases with a single isocenter. However, Lai et al.^32^ assert that Catalyst HD may not serve as a substitute for non‐coplanar CBCT (NC‐CBCT) in correcting non‐coplanar setups for single‐isocenter SRS for single and multiple brain metastases. This conclusion stems from their observation of variations between the two systems, reaching up to 1.5 mm and 1.1° in LNG and yaw directions, respectively. Moreover, the study revealed that only 35.71% of the absolute set‐up error detected by Catalyst HD and NC‐CBCT was within the tolerance of 0.5 mm/0.5°, in both systems simultaneously.

On the other hand, Lai et al.^32^ exclusively present accuracy comparisons between Catalyst HD and NC‐CBCT for couch rotations ≤45°. This limitation arises from potential collisions between the couch and CBCT system at larger couch angles. These authors declare that a further investigation of the use of planar kV/MV images for couch rotations > 45° must be done. This study partially addresses this lack of knowledge: the feasibility of the SGRT system to verify non‐coplanar set‐up for any table angle is compared with the verification of the set‐up performed by the MV image system (Figure 4a). The LNG, LAT, or yaw corrections for any table rotations were not significantly different, except for LNG shifts at 65° (*p* = 0.04) and 75° (*p* = 0.03) table angle position; LAT shifts at 65° (*p* = 0.03) and 270° (*p* < 0.001) table angle position, and yaw rotation at 30° (*p* = 0.02) table angle position. The maximum differences between AlignRT and the MV image were −0.9 ± 1.4  and 0.5 ± 1.4 mm in the LNG and LAT directions, respectively, for a table position of 65°, and −0.4° ± 0.4° of yaw for a table position of 30°. This data are comparable to those reported by Zhang et al.^38^ and Wiant et al.^27^ for table positions close to ± 90°.

Regarding intrafraction control, Wiant et al.^27^ demonstrated loss of < 0.2 mm for 72 table‐gantry configurations using an anthropomorphic head phantom. Therefore, the use of non‐coplanar beams does not cause a loss of accuracy in intrafraction control because of the use of smaller monitoring ROIs obtained by propagation and/or possible occlusion of some cameras. In fact, the novel use of 1 s of “maximum misalignment time” compensates for the false deviation detected by the SGRT system when a camera POD was obstructed by the gantry. Our results (Figure 6) show that intrafraction movements were on average < 0.1 mm and 0.1° for each table position, which are comparable to the results of Han et al.^51^ who analyzed 11 patients immobilized with the Klarity open‐face mask and treated with coplanar beams. The longitudinal direction was again where the largest movement was found as reported in other works.^31^, ^33^ Covington et al.^31^ found similar results, but the magnitude of LNG shift was up to −0.64 mm at 270° couch angle, probably because they used a patient threshold of 1 mm, rather than 0.3 mm like we did, or even due to manual rather than gated beam control. Moreover, in other work,^33^ they indicated that the decreased localization accuracy in the longitudinal direction may be attributed to the camera configuration. On the other hand, the thermal drift of the optical system used in SGRT can increase the uncertainty of the patient's movement monitoring.^52^ The SGRT system can exhibit a drift of 0.3 mm in the VRT direction after 10 min of monitoring.^33^, ^38^, ^41^ This effect does not seem to affect our intrafractional control, as the use of AlignRT from the beginning of the treatment ensured enough warm up time of the cameras.

This is the first time in the literature where thresholds of 0.3 mm and 0.3° for control beam delivery have been reported, deviating from the typical thresholds commonly reported at 1 mm and 1°. These thresholds are compatible with the accuracy that AlignRT can achieve as other authors have shown in their studies using head phantom and couch rotations: Wiant et al.^27^ demonstrated a similar accuracy to ExacTrac system; Li et al.^41^ obtained an accuracy of 0.1 ± 0.1 mm of LAT and LNG displacement; Oliver et al.^25^ determined the accuracy of a SGRT system as ≤ ± 0.25 mm and ± 0.20° However, the use of very low thresholds can increase treatment time and therefore greater intrafractional motion. Han et al.^51^ documented an increase in treatment time of 9.4% when using 1 mm/1° thresholds. In our center, the total treatment time was 30.63 min (SD = 9.03 min) using the 0.3 mm/0.3° threshold, which did not increase the time required for this type of treatment previously carried out with the BrainLAB Exac Trac. This agrees with times reported by other studies using similar intrafractional control techniques.^53^ The use of a 1‐s “maximum misalignment time” allowed treatment to continue even when these thresholds were exceeded, thus avoiding numerous interruptions due to occlusion of some of the PODs. Dosimetrically, intrafractional motion during frameless SRS can cause underdosing of target volumes, poorer local control, and increased healthy brain irradiation, highlighting the importance of rigorous intrafractional control.^54^

It is imperative to highlight that, given the SGRT system uses the surface of the patient's face as a surrogate for tumor motion and can report a false increase in the magnitude of translational offsets at non‐coplanar couch position,^31^, ^37^ it is not possible to rely solely on surface monitoring for tumor position verification and intrafraction control. In this regard, this study has a limitation: the accuracy of AlignRT has not been assessed in all the six DoF for table angles other than zero. Further investigations will be conducted to determine the collision‐free space for different table positions where orthogonal kV/kV or kV/MV images can be acquired. The results will then be compared with those obtained using with AlignRT. Another limitation is that data are restricted to one SGRT system with a particular environmental factors such as ambient lighting or temperature fluctuations that could contribute to differences in performance between systems, although the system was installed and operating according to manufacturer's specifications. In addition, the fact that the sample size of patients with Qfix mask is smaller than with Klarity mask may affect the precision of the estimates made.

## 5 CONCLUSION

This work presents the feasibility of using the AlignRT SGRT system in conjunction with the IGRT system of a TrueBeam STx accelerator for use in non‐coplanar beam fSRS treatments. Using real patient data, AlignRT could achieve a precision of 0.5 mm/0.1°, except in longitudinal direction and yaw rotation, where the precision is slightly lower, and a real‐time patient monitoring for any table angle with an accuracy of better than 0.1 mm/ 0.1°, with a similar occupancy time to that used in the center before the use of SGRT.

The Encompass SRS Fibreplast mask or Klarity Blue mask, together with the Qfix Encompass™ immobilization system, provide a suitable fixation. This ensures the precision required for fSRS treatments while providing an adequate surface for monitoring with AlignRT.

## AUTHOR CONTRIBUTIONS

**Patricia Sánchez‐Rubio**: Conceptualization, investigation, writing—original draft, visualization. **Ruth Rodríguez‐Romero**: Data curation, writing—review & editing. **María Pinto‐Monedero**: Formal analysis; writing—review & editing. **Luis Alejo‐Luque**: Resources, validation, writing—review & editing. **Jaime Martínez‐Ortega**: Writing—review & editing.

## CONFLICT OF INTEREST STATEMENT

The authors declare no conflicts of interest.

## Supporting information



Supporting Information
