# Supplementary material for: New findings on clinical experience on surface‐guided radiotherapy for frameless non‐coplanar stereotactic radiosurgery treatments
Source: J Appl Clin Med Phys. 2024 Sep 17;25(12):e14510. doi: 10.1002/acm2.14510 (PMC11633809; doi:10.1002/acm2.14510)
Supplement: Supplementary file 1 — Supporting Information [file ACM2-25-e14510-s001.docx]

**Supplementary Material Table1.** Statistical description of the differences between AlignRT – MV image for LNG, LAT displacements and yaw rotation.

|  |  | **AlignRT – MV LNG (mm)** | | | | **AlignRT – MV LAT (mm)** | | | | **AlignRT – MV YAW (°)** | | | |
| --- | --- | --- | --- | --- | --- | --- | --- | --- | --- | --- | --- | --- | --- |
| ***Couch angle*** | ***n*** | ***Mean*** | ***SD*** | ***Max*** | ***Min*** | ***Mean*** | ***SD*** | ***Max*** | ***Min*** | ***Mean*** | ***SD*** | ***Max*** | ***Min*** |
| **T000** | 3 | 1.0 | 1.9 | 3.1 | -0.2 | 1,1 | 1,1 | 2,1 | 0,0 | 0,3 | 0,5 | 0,8 | 0,0 |
| **T010** | 3 | 0,1 | 2,1 | 2,4 | -1,7 | -0,3 | 0,2 | -0,1 | -0,4 | 0,1 | 0,5 | 0,5 | -0,5 |
| **T025** | 5 | 0.0 | 0.6 | 0.7 | -1.0 | -0.4 | 0.2 | -0.2 | -0.6 | -0.1 | 0.5 | 0.3 | -0.8 |
| **T030** | 11 | -0.3 | 0.9 | 1.8 | -1.5 | -0.2 | 0.6 | 1.2 | -0.9 | -0.4 | 0.4 | 0.5 | -1.0 |
| **T045** | 13 | -0.3 | 0.6 | 1.1 | -1.2 | 0.0 | 0.7 | 1.3 | -1.1 | 0.0 | 0.8 | 1.8 | -1.2 |
|  |  |  |  |  |  |  |  |  |  |  |  |  |  |
| **T065** | 13 | -0.9 | 1.4 | 1.0 | -4.7 | 0.5 | 1.4 | 5.2 | -0.1 | -0.1 | 0.6 | 0.8 | -1.3 |
| **T075** | 16 | -0.5 | 0.6 | 0.9 | -1.7 | 0.1 | 1.0 | 2.0 | -2.6 | 0.0 | 0.5 | 1.0 | -0.9 |
| **T090** | 17 | -0.4 | 1.0 | 1.6 | -1.9 | 0.2 | 0.9 | 2.4 | -2.0 | -0.1 | 1.0 | 1.6 | -2.4 |
| **T270** | 25 | 0.0 | 0.6 | 1.5 | -1.0 | 0.4 | 0.5 | 1.0 | -0.7 | -0.2 | 1.2 | 2.1 | -4.9 |
| **T285** | 8 | -0.3 | 0.7 | 0.5 | -1.3 | 0.4 | 0.5 | 1.3 | -0.2 | 0.1 | 0.4 | 0.4 | -0.5 |
|  |  |  |  |  |  |  |  |  |  |  |  |  |  |
| **T300** | 14 | -0.3 | 1.4 | 1.6 | -4.1 | 0.4 | 1.0 | 2.3 | -1.4 | -0.1 | 0.6 | 0.6 | -1.5 |
| **T315** | 12 | -0.2 | 0.5 | 1.1 | -1.0 | 0.3 | 0.8 | 1.9 | -1.1 | 0.2 | 2.0 | 4.4 | -4.1 |
| **T330** | 11 | 0.0 | 1.0 | 2.1 | -1.7 | 0.3 | 0.6 | 1.3 | -0.6 | -0.1 | 0.5 | 0.4 | -1.4 |
| **T340** | 2 | 0.2 | 0.5 | 0.5 | -0.2 | 0.2 | 0.0 | 0.2 | 0.2 | -0.2 | 0.7 | 0.3 | -0.7 |
| **T350** | 4 | 0.0 | 0.4 | 0.3 | -0.5 | 0.2 | 0.4 | 0.8 | -0.1 | -0.1 | 0.4 | 0.2 | -0.6 |

n: number of lesions
